# Exploring exercise adherence and quality of life among veteran, novice, and dropout trainees

**DOI:** 10.3389/fspor.2023.1293535

**Published:** 2023-11-20

**Authors:** Maor Gabay, Ofer Levi, Simona Petracovschi, Cristian Negrea, Marius Matichescu, Mihaela Oravitan

**Affiliations:** ^1^ Faculty of Physical Education and Sports, West University of Timișoara, Timisoara, Romania; ^2^ Department of Physical Education and Sports, Kaye Academic College of Education, Be’er Sheva, Israel; ^3^ Department of Mathematics and Computer Science, The Open University of Israel, Raanana, Israel; ^4^ Faculty of Sociology and Psychology, West University of Timișoara, Timișoara, Romania

**Keywords:** exercise, adherence, training, dropout, physical activity, habit

## Abstract

**Introduction:**

The purpose of this study was to identify and reveal the different contexts, variables, and factors that may influence adherence to physical activity among veteran, novice, and dropout trainees, such as the frequency of the weekly training units, the trainees preferred type of exercise, the purpose of the physical activity, and the relationship between support and supervision by fitness instructors and professionals. This study also examined the relationships between trainees, seniority and the strength of the habit and adherence to physical activity, the effects of personal variables such as age and gender on adherence to physical activity, and how the seniority and adherence of trainees may affect their quality of life.

**Methods:**

A total of 460 participants drawn from the broader Israeli exercise community, which encompasses a diverse range of individuals within the general adult population, were engaged in this study. These participants included seasoned exercisers, individuals who had recently initiated exercise routines, and those who had previously engaged in physical activity. Each participant completed a comprehensive set of questionnaires, including the Self-Report Habit Index, the Exercise Adherence Rating Scale, and the World Health Organization (WHO) Quality of Life Scale. In addition to the questionnaire responses, demographic data and inquiries concerning their physical activity were also collected.

**Results:**

The results show that the average frequency of the weekly training units of veteran trainees was significantly higher than that of novice trainees: 3.95 and 2.93, respectively (*p < *0.0001). We found no significant differences between novice and veteran trainees regarding their training goal preferences (*p = *0.07). Veteran trainees who had been in training for more than a year appeared to have higher self-efficacy since 31.16% reported receiving no supervision compared to 16.67% among novice trainees. In addition, people over 45 appear to have more health-related goals compared to their younger counterparts (*p *< 0.001). The quality of life scores of the trainees was related to their seniority in physical training, but only by a small magnitude (*R*^2^ = 0.06), *p* < .001). Those who trained in resistance training showed the greatest rate of adherence in relation to flexibility and aerobic training (*p *< 0.001), and women preferred more body toning and had more weight loss goals than men (*p *< 0.001).

**Discussion:**

The results indicate that there are central and important factors that may affect adherence to physical activity, and that all these aspects must be taken into account when planning a training program or when there is a desire to maintain or increase adherence to physical activity. The research findings indicate that the main factors that can influence adherence to physical activity are identifying and increasing the trainee's self-efficacy, maintaining weekly training units with sufficient frequency to form a habit and incorporating resistance training into the training regime, as resistance training has shown high levels of adherence. Moreover, it seems that people with different degrees of experience in physical training have distinct and varied training goals, and there is no one goal that fits all. In addition, specific factors such as age and gender must also be taken into account, because the age and gender of the trainees may significantly affect the goals of physical training.

## Introduction

Numerous studies have underscored the pivotal role of regular physical activity in the management and prevention of noncommunicable diseases. Adherence to the physical activity guidelines set forth by the World Health Organization (WHO) has demonstrated the potential to substantially mitigate the risk of premature mortality by 20%–30%, while also contributing to a reduction in prevalent health issues such as cardiovascular diseases, diabetes, and dementia (1). Moreover, many studies that investigated the effects of physical activity on psychological discomfort, anxiety, and depression discovered that its effects may be comparable to those of medicine and psychotherapy (2). In addition, exercise adherence has a beneficial effect on physical function, pain, and chronic diseases (3). However, even though the benefits of having an active lifestyle are so well known, a major health concern is that a sedentary lifestyle and lack of physical activity are still so prevalent (4). Therefore, physical inactivity may even be considered a pandemic due to its prevalence and societal repercussions (3). Recent global estimations reveal a concerning prevalence as 1.4 billion adults, equivalent to 27.5% of the global adult populace, are failing to meet the recommended thresholds for physical activity, which are fundamental for the enhancement and safeguarding of their health (1). In the sphere of physical activity, the concept of adherence assumes a paramount significance, representing an individual's steadfast commitment to a structured training regimen (5). The online *Oxford Dictionary of Sport Science and Medicine provides* a definition of exercise adherence, describing it as “maintaining active involvement in physical activity.” It further emphasizes that individuals with strong exercise adherence continue their participation in physical activity, even in the face of opportunities and pressures to withdraw (6). However, it's important to note that the definition of exercise adherence varies significantly across the literature, comprising four distinct measures: completion, attendance, duration adherence, and intensity adherence (7). In the realm of adherence assessment, various studies have adopted attendance as a pivotal criterion (8, 9), with indications that irregular attendance patterns can effectively identify individuals who may require adherence counseling (10). The complexity of participation and adherence to physical activity is further accentuated by the diverse array of factors influencing an individual's engagement, encompassing motivation, abilities, preferences, self-efficacy, social dynamics, environmental conditions, and policy considerations (11), along with social context, habit and past behavior, professional support, motives and barriers. In addition to personal characteristics such as gender, age, and level of seniority at the fitness facility affect participation (12).

Moreover, personal characteristics such as obesity, blue-collar status, and smoking have been associated with decreased adherence to an exercise program and an increased dropout rate. In addition, exercise programs have many variables that need to be taken into account that may also affect adherence such as the activity type, intensity, flexibility, and the cost of the program (13). Burnet et al. (14) identified specific physical training variables such as Frequency, Intensity, Time, and Type of activity (FITT) that may influence exercise adherence among different populations. Furthermore, beyond general physical activity, engagement in organized physical activity such as “exercise” has decreased, as evidenced by a drop in people's involvement in regular and scheduled physical activity in recent years (15). Moreover, a study of 5,240 members of a fitness club in Brazil found large abandonment rates, as 63% of the participants had abandoned their membership in the first quarter, and after a year, the abandonment rate reached an alarming 96% (16). Another study conducted in Spanish gyms showed abandonment rates of about 51% in a 1-year period (17). Still another study found that only 37% of participants reported regular adherence during the first year of their gym membership (18), while a recently published retrospective observational cohort study conducted in two gyms found adherence rates of only 11% and 19% (3).

According to these data, adherence to physical activity in general is problematic, particularly in high-income countries, where the rates of inactivity are twice as high as in low-income countries (1). In addition, there is evidence of low adherence rates in institutions such as fitness clubs and gyms (3, 16, 18). which play an important role in enabling recreational sports and daily physical activity to take place (19), promoting physical activity for individuals and groups, and providing a wide context for physical activity (20). On a global scale, the fitness and health sector is growing rapidly on a global scale. According to data, there are now over 150,000 fitness and health facilities worldwide, with over 140 million members, and the industry is worth $77.5 billion (21). Currently, the United States leads the world's gym market with over 30,000 establishments, followed by Brazil with approximately 24,000 units (22). According to data from Europe in 2018, the average number of members per fitness club increased at a rate of 7%, while the market as a whole was increasing at a rate of 3.2% based on the number of clubs (23). By using fitness centers, people of all body types may exercise their bodies in a variety of ways, use a wide range of fitness equipment, and receive personalized advice from fitness professionals (24). Fitness centers stress and encourage many individuals to have healthy lives by exercising and receiving instruction (25). However, despite this data, it is clear that the fitness business suffers from high churn and low member loyalty, as well as the fact that between 19% and 24% of consumers discontinue partaking in sports activities after leaving the sports center (26). Aside from the fact that leaving gyms and sports centers is a major economic worry, this has the potential to become a global problem in terms of physical inactivity because sports centers are the primary drivers of such activity, creating a major public health concern (17).

Accordingly, the purpose of this study was to identify and reveal the various contexts, variables, and factors that may affect adherence to physical activity among veteran, novice, and dropout trainees and to understand whether this adherence may also affect their quality of life.

## Overview and hypotheses

The purpose of this study was to understand the differences between experienced and novice trainees, and people who had exercised in the past, but who were not exercising currently. In addition, we sought to investigate and identify the variables, contrasts, and contexts that may affect exercise adherence, including the frequency of weekly training units, type of training preferred by the trainee, purpose of the training, and the relationship between support and supervision by the fitness instructors. Moreover, we examined the relationships of seniority with habit strength and adherence to physical exercise, the effects of individual characteristics such as age and gender on exercise adherence, and how trainees' seniority and adherence may affect their quality of life.

It is believed that the frequency of exercise affects adherence rates. Rodrigues et al. (15) showed that a weekly frequency of two workouts per week led trainees to maintain a future routine. Kaushal and Rhodes (27) found that in order to establish physical exercise habits, it was necessary to exercise at least four times a week and for a duration of about 6 weeks, while Clavel San Emeterio et al. (17) showed that a frequency of more than eight times a month is a parameter that significantly reduced the chance of retirement. Based on these findings, and leveraging the dataset at our disposal, our initial conjecture revolves around veteran trainees, delineated as individuals possessing extensive experience, spanning over 12 months, who consistently uphold a training frequency surpassing the threshold of three sessions per week. An additional supposition underlies our analysis, postulating that the training frequency of veteran trainees surpasses that of their novice counterparts. Consequently, we formed the following hypothesis:

**Hypothesis 1**. *Trainees who adhere to physical exercise for more than 12 months, training with an average weekly frequency of above 3 sessions per week and will develop a stronger habit for physical exercise compared to novice trainees*.

The type of exercise training may also have an effect on adherence rates. Robison and Rogers (13) found that approximately 50% of individuals who start an aerobic exercise program will stop within the first six months. van der Vlist et al. (28) concluded that the tedious nature of endurance exercise makes it difficult for many people to establish a healthy exercise habit. Lee et al. (29) found that in overweight teenagers, a group who participated in aerobic training reported more boredom and less pleasure than a group involved in resistance training. Moreover, Picorelli et al. (30) showed that among older women, adherence rates to strength training were higher than to aerobic training. The reasoning behind the assumption that women will typically engage in more aerobic activity than males is based on research by Dworkin (31), who found that women preferred aerobic activities over weight training because they perceived that such activity allowed them to not only build strength but to maintain a feminine appearance. Thus, we believe:

**Hypothesis 2**. *The rates of adherence to physical exercise will be higher for those who engage in resistance training than for those in aerobic training, flexibility training, or other activities. In addition, in general and regardless of the level of adherence, women will show more engagement in aerobic activity than in resistance training compared to men*.

Age and gender seem to affect the purpose of exercise. According to research by Kilpatrick et al. (32), among students with an average age of 22 years, concerns about beauty and weight control were important driving forces behind physical exercise. Caglar et al. (33) found that appearance-related motives were important for young adults, and Soekmawati et al. (25) showed that adults are motivated by aesthetic goals such as appearance and weight management.

In addition, women not only want to maintain their health (with an emphasis on maintenance and not improvement), but they also wish to increase their attractiveness and improve their appearance (25). Kilpatrick et al. (32) found that female college students had more concerns about their body weight than males, and Koivula (34) showed that women rated appearance as more important than men. Soekmawati et al. (25) reported that women were 2.4 times more likely to choose appearance-related goals as a motivation for physical exercise, and had greater concerns greater concerns about their body weight than men. Anić et al. (35) showed that women with a high BMI exercised to lose weight and that it was their body composition that affected their satisfaction and droves them to prefer weight management as their main motivation for exercise. In contrast, men were more motivated by social recognition, competition, challenge, strength, and endurance, and saw physical exercises as a means of achieving these ego-related results (25).

The assumption that the older the exercisers are, the more likely they are to pursue health-related goals stems from the findings of Lübcke et al. (36) who, in interviews with subjects aged 65–81, demonstrated that, over time, physical exercise became for them an investment in health and social activity. Trujillo et al.'s (37) findings indicate that elderly people are more concerned about health outcomes than younger people. Therefore, we propose that:

**Hypothesis 3**. *There will be a positive correlation between the age and gender of the exerciser and the purpose of the physical exercise, and as age increases, training goals will be more related to health related than to aesthetics. In general, women's goals will be more aesthetic, especially in relation to toning the body and losing weight, compared to men*.

The type of motivation trainees have affects their exercise goals. Ortís et al. (38) showed that external motivation was dominant during the early stages of behaviorial change in exercise, whereas at later stages and for long-term maintenance, internal motivation dominated. Furthermore, intrinsic motivation was positively associated with exercise adherence, and individuals with higher levels of intrinsic motivation reported higher levels of physical activity persistence and adherence over time (39). Thus, we hypothesize that:

**Hypothesis 4**. *Experienced exercisers will prioritize goals related to improving physical fitness and health over goals of visibility and aesthetics because they likely have developed more internal motivation compared to beginners who depend more on external motivation*.

It would seem to make sense that trainees would benefit from guidance and instruction in the early days of their training, and that the longer they exercise, the less dependent they would become on this external support. Sperandei et al. (16) observed that adherence to physical activity in unsupervised programs was low, adding that the findings of their study revealed that more than half of gym members did not complete 3 months of active participation and that there was a less than 5% chance that a person would remain active for more than a year. Thus, receiving support from physical fitness instructors may create favorable conditions for the promotion of and adherence to long-term physical activity (15). According to Klain et al. (40), subjects who had personal trainers had more self-determined forms of regulation and were more adherent to physical exercise. Teixeira and Palmeira (41) stressed the importance of fitness professionals who understand how to build support for trainees in order to improve their adherence and psychological well-being. Based on these findings, we expect to find that:

**Hypothesis 5**. *Most veteran trainees (over a year) received guidance and support in the past, but do not currently receive such guidance and, thus, are more independent*.

Lastly, because physical activity has a great effect on human health, both physiologically (42) and mentally (43), and this greatly impacts a person's well-being and quality of life (44), our last hypothesis is:

**Hypothesis 6**. *People who persist and adhere to long-term physical activity have a better quality of life*.

## Materials and methods

### Participants and procedure

812 Israeli participants were recruited through social media and instant messaging apps. After giving informed consent, volunteers completed a Hebrew online research questionnaire about physical activity and quality of life. Some of the instruments included in the questionnaire already existed in Hebrew, whereas others were translated from English into Hebrew using the back translation method (45, 46). The Ethics Committee of West Timisoara University approved the questionnaire and research protocol (15227/7.03.2023). We excluded data from 352 participants, as 24 were screened out based on the first question in the study, and 328 failed to complete the responses in the questionnaires or filled them in carelessly or illogically and/or did not meet the inclusion criteria of the study*.* In total, the final sample comprised 460 participants with a mean age of 37.03 (SD = 11.71 years), and 53.7% were female. The BMI for the female participants (*N* = 247) was 23.66 (sd = 3.77), and for the males (*N* = 153), it was 25.93 (sd = 3.83). For the total sample (*N* = 460), the BMI was 24.71 (sd = 3.96), suggesting that the sample overall was of normal weight (47). The majority of the participants had a post-secondary education, with 69.80% having an academic education, and 71.74% of the participants reported that they worked fulltime. Table 1 presents the remainder of the participants' characteristics, and their descriptive data are displayed in Table 2.

**Table 1:** Characteristics of participants.

	Male (*N* = 213)	Female (*N* = 247)	All (*N* = 460)
	Mean (±SD)	Mean (±SD)	Mean (±SD)
Age	36.85 (±10.87)	37.18 (±12.41)	37.03 (±11.71)
Body weight (kg)	81.58 (±13.99)	63.12 (±11.12)	71.66 (±15.54)
Body height (cm)	177.14 (±6.23)	162.45 (±10.83)	169.25 (±11.60)
BMI	25.93 (±3.83)	23.66 (±3.77)	24.71 (±3.96)
Seniority level adherence to exercise and/or physical activity (years)	8.73 (±9.28)	7.85 (±8.76)	8.25 (±9.01)
Seniority level adherence to exercise and/or physical activity (months)	1.58 (±2.69)	1.16 (±2.28)	1.36 (±2.49)
Training sessions per week	3.72 (±1.89)	3.14 (±1.81)	3.41 (±1.87)

**Table 2:** Descriptive data.

Characteristic		Percentage
Education	Less than secondary education	.4
	Secondary education	29.8
	Academic education	69.8
Employment	Unemployed	10.7
	Part time	17.6
	Full-time	71.7
Marital status	Single	36.5
	Married	55.0
	Divorced/separated	7.2
	Widowed	1.3
Supervision	No supervision at all—trains completely independently	36.7
	Supervised by a shift instructor in the gym who is responsible for all the trainees	15.9
	Online support (online trainer)	5.7
	Training in small groups (3–6 participants)	25.9
	Personal training (one-on-one)	15.9
Preferred type of training	Aerobic training	25.9
	Resistance training	54.6
	Flexibility training	9.1
	Other	10.4
Goals	Health	29.8
	Toning and weight loss	31.5
	Increase in muscle mass	17.2
	Improving physical fitness	21.5

### Instruments and measures

For inclusion in this study, participants were required to be: (1) at least 18 years of age, (2) Hebrew speaking and with no difficulty in reading and filling out the questionnaires, and (3) either actively exercising at the moment or they had done so in the past. Accordingly, 812 exercisers answered the first question, which explained that the study was intended for people who had exercised and engaged in physical exercise in the past or who were currently engaged in an exercise regime. The purpose of this question was to initially identify the relevant subject population for the study. Of the respondents, 168 (20.96%) reported that they had exercised in the past, but were not currently exercising; 620 (76.35%) reported that they were currently exercising, whereas 24 (2.96%) reported that they were not currently exercising and had not exercised in the past. This last group was excluded from answering any further research questions.

### Habit strength

The Self-Report Habit Index (SRHI) is an instrument for assessing habit strength. It consists of a basic question (stem) with variable responses that the researcher can choose and formulate according to his specific requirements, for example, “[Behavior X] is something…”, with 12 items that assess aspects of the habit. In this study, the basic question was “Physical exercise is something that…” followed by e.g., “I do automatically”, “I do frequently”, “I do without having to consciously remember”, and so forth (see Table 3 for the specific statements). The participants were asked to rate their degree of agreement using a 5-point Likert scale. After this, internal reliability test of the scale was carried out (*α* = 0.92). Finally, an average of the items was compiled to assess the strength of the habit (48).

**Table 3:** SRHI questionnaire *t*-test.

Item	Beginners (*n *= 54)		Veterans (*n *= 337)		*T*
	Mean	*SD*	Mean	*SD*	
1. I do frequently	3.78	1.14	4.61	.67	−7.51***
2. I do automatically	3.13	1.16	3.96	1.05	−5.33***
3. I do without having to consciously remember	2.74	1.15	3.31	1.32	−2.96*
4. That makes me feel weird if I do not do it.	3.24	1.31	4.33	.94	−7.42***
5. I do without thinking	2.89	1.19	3.35	1.26	−2.62*
6. That would require effort not to do it	2.76	1.24	3.36	1.22	−3.28**
7. That belongs to my (daily, weekly, monthly) routine	3.95	1.02	4.61	.59	−6.72***
8. I start doing before I realize I’m doing it	2.55	1.07	2.87	1.22	−1.99
9. I would find hard not to do.	3.04	1.23	4.21	1.06	−7.34***
10. I have no need to think about doing.	2.84	1.23	3.37	1.26	−2.97**
11. That's typically “me”	3.32	1.16	4.23	.92	−6.56***
12. I have been doing for a long time	3.28	1.34	4.61	.61	−12.06**

* *p* < 0.05.

** *p* < 0.01.

*** *p* < 0.001.

### Exercise adherence, helps and hindrances

In order to capture the exercise adherence of the participants and the factors that might help or hinder them, we used the Exercise Adherence Rating Scale (EARS) (49). Meade et al. (50) demonstrated that the EARS is a robust measure of adherence, with good face validity and comprehension.

It is a three-part self-report index that assesses and measures adherence to prescribed home exercise: The first section requires participants to document the exercise prescription provided to them by their healthcare provider, the second section measures the level of adherence using six items, and the third section measures ten items (factors that help or hinder) that affect exercise adherence. In this study, we did not require participants to complete the first section. Measurement was performed only according to data provided in the other sections (*α* = 0.88). The EARS includes six adherence items and ten additional items of helps and hindrances. The score of each participant is the sum of the responses of all 16 items using a 5-point Likert scale.

### Quality of life

In order to assess the quality of life of the study participants, we used the WHOQOL: Measuring Quality of Life questionnaire, a cross-culturally applicable quality-of-life assessment (51) that was obtained from the WHO website. This questionnaire probes how a subject feels about his quality of life, health, and other areas of life. It consists of 26 questions in total that are rated on a 5-point Likert scale (*α* = 0.91). A Hebrew translation of this questionnaire was done independently by the Department of Behavioral Sciences at the Ben-Gurion University of the Negev, Israel.

### Statistical analyses

Data analysis was performed using IBM SPSS version 23.0. We utilized F-tests to determine the variance ratio between two samples, t-tests to find the mean difference between two independent samples with equal variance, and ANOVA and post-hoc tests for homogeneity. Chi-square tests were employed to see differences between gender, and veterans and novice trainees, and a Pearson chi-square determined the relationship between the age and gender of trainees and their training goals. Accordingly, a significance level of *p* = 0.05 was determined, and the effect size was evaluated for each test. Internal reliability of the questionnaires was performed using Cronbach's alpha.

## Results

In order to examine Hypothesis 1, a confidence interval was first evaluated first, for the weekly training frequency among the 337 participants who had trained for more than 12 months with the following results: ${\bar{x}}_{n}=3.947,\cdot {\bar{s}}_{n}=1.563$, with a confidence level of 0.95. The formula ${\bar{x}}_{{}_{n}}\mp t_{n-1,0.975}\frac{\bar{s_{n}}}{\sqrt{n}}$, produces a CI of [3.779,4.114] for which there is a 95% probability that the actual frequency value is inside. Next, a single tail *t*-test was carried out for the null hypothesis H_0_: *µ* = 3, vs. the alternative hypothesis H_1_: *µ* > 3. The null hypothesis was rejected with *p* < 0.00001, strongly supporting H_1_. Additionally, a *t*-test comparing those who were currently training for more than 12 months (*N* = 337) to those who had trained for 12 months or less (*N* = 54) revealed a significant statistical difference between the two groups regarding the number of weekly training units they had performed: *t*(389) = −4.46, *p *= .000. Accordingly, the veteran trainees (more than 12 months) had an average score of 3.95 (sd = 1.56) weekly training units compared to the novice trainees whose average was 2.93 (sd = 1.55). Cohen's d value for the effect size was 0.66, indicating a medium effect.

Regarding the strength of the habit of physical exercise, the results of the SRHI questionnaire (52) that assessed habit strength were separated into two samples—one for those who had been training for over 12 months and the other for those training for at most 1 year. A chi-square test for the variance ratio between the two samples for each of the 12 items of the questionnaire showed variance equality for items 1–4, 7, 9, 11 and 12 (see Table 3) with *p *< 0.05. Therefore, a *t*-test determined the mean difference between two independent samples with equal variance applied to these items, as well as for two samples with unequal variances to the rest of items. See Table 3 for a summary of these results.

The EARS was evaluated for each of the participants, giving an individual adherence score in the range of (16, 80), in which 16 represents the lowest adherence rating and 80 the highest.

A statistical summary of the CIs for the adherence score in each training category is shown in Table 4. An *F*-test was used to measure for variance homogeneity of *p *< 0.001 between the four training categories. ANOVA results show a significant difference between the four categories with *F* (3,456) = 23.49*, p *< 0.001, and an effect size of 0.14 using the eta measure. A *post-hoc* test for homogeneous subsets with a 0.05 level of significance resulted in the following subsets: (1) Flexibility training, (2) Aerobic training + other, and (3) Resistance training + other. The resistance trainees' adherence was significantly higher than that of the aerobic (*p *< 0.001) and flexibility trainees (*p *< 0.001), whereas the difference between the resistance trainees’ adherence and the “other” category was not significant (*p *= 0.34).

**Table 4:** Adherence rating (EARS).

	*N*	Mean	Std. deviation	95% confidence interval for mean	
				Lower bound	Upper bound
Aerobic training	119	60.91	11.17	58.88	62.94
Resistance training	251	66.46	9.08	65.34	67.59
Flexibility training	42	53.36	12.53	49.45	57.26
Other	48	63.52	10.92	60.35	66.69
Total	460	63.53	10.92	62.53	64.53

The fourth hypothesis was investigated using a chi-square test. While a gender difference in resistance and aerobic exercise preferences was identified, it was not demonstrated to be statistically significant: χ^2^ = (1, *N* = 370) = 1.76, *p* = 0.18.

Two independent Pearson chi-square tests were used to determine the relationship between the age and gender of the trainees and their training goals (Hypothesis 3). The first test between the age groups (age ≤45 or age >45) and the training goal was significant with a medium effect size (*χ^2^* =^ ^(1, *N *= 460)^ ^=^ ^19.97, *p *< 0.001, phi = 0.22), showing that 48% of the participants in the older group (age >45, *N* = 99) preferred health goals over others, in contrast to only 24.6% of the younger group (age ≤ 5, *N* = 361). The second test, between gender and training goals, also had a significant result, with a medium effect size (*χ^2^* =^ ^(1, *N*^ ^=^ ^460)^ ^=^ ^18.97, *p*^ ^<^ ^0.001, phi^ ^=^ ^0.21).

In testing the expectation that experienced exercisers will prioritize health goals and goals related to improving physical fitness over those of visibility and aesthetics (Hypothesis 4), a distinction between health and aesthetic goals (body toning and shaping, visibility, and muscle mass increase) was made. A chi-square test conducted between novice (less than a year of experience) and advanced (more than a year of experience) participants who reported that they were currently training, using a chi-square yielded results of: *χ^2^* = (1, *N* = 391) = 3.19, *p* < 0.07.

In testing the fifth hypothesis, the results showed that 28.19% of the veterans reported that they were being given instruction and guidance, 40.65% had received support in the past, but 31.16% had not received any support at all. In contrast, 44.44% of the novices reported that they were currently receiving extra support, 38.89% had received support in the past, while 16.67% had not. Along with these data, it is important to note that a chi-square yielded the following results with a small effect size: (*χ^2^ *=* *(2, *N *=* *391)* *=* *7.43, *p *<* *0.024, phi* *=* *0.14).

To examine the quality of life in relation to the level of seniority of the trainees (Hypothesis 6), we used the WHOQOL questionnaire. A regression analysis indicated that there was a significant effect between the seniority level and quality of life: *F*(1, 457) = 30.87, *p* < .001. The adjusted *R^2^* indicated that only 6% of the variance in the quality of life could be explained by the seniority level, which was shown to be a statistically significant predictor of quality of life (*t* = 5.56, *p* < .001). The regression model suggested that each increase in seniority level was related to a 0.37 mark improvement in the quality-of-life score. The regression equation for this model was: quality of life; *score* = 96.97 + (0.37 X seniority).

## Discussion

The purpose of this study was to comprehensively investigate the array of contextual, variable, and situational factors that could potentially influence exercise adherence across distinct categories of individuals, including those with varying levels of experience in physical activity, ranging from seasoned participants to beginners and individuals who had discontinued their exercise routines. A secondary aim was to examine whether this adherence might be associated with discernible effects on their overall quality of life. In accordance with this research objective, our findings offer substantial support for our initial hypotheses. Specifically, the results highlight a considerable and statistically significant distinction between veteran trainees and novices. Furthermore, our data reveals that veteran trainees consistently engage in training sessions exceeding a frequency of three times a week, with an observed practical frequency approaching four times weekly. The frequency of a specific behavior assumes a crucial role, with repeated practice culminating in the establishment of a “routine behavior.” This process integrates the behavior as an integral component of one's personal identity (53) and may illustrate the profound influence of consistent exercise habits on one's self-perception and daily routines. Additionally, it is prudent to consider that certain individuals engaging in exercise may modulate and oversee their exercise frequency based on their physiological capacities and adherence to exercise prescriptions in accordance with health organizations[such as the American College of Sports Medicine (ACSM) guidelines]. The adjustment in exercise frequency can be self-regulated or carried out under the supervision of a healthcare professional. This approach is warranted by prior research demonstrating that exercise frequencies of up to twice weekly can elicit improvements in aerobic capacity among individuals with lower fitness levels. However, it is essential to recognize that when aerobic capacity surpasses the threshold of 50 ml/kg/min, a minimum exercise frequency of at least three times weekly is deemed necessary (54). Expanding on this discourse, and as mentioned above, it is plausible that individuals across diverse fitness levels adhere to the guidelines and directives prescribed by health organizations. For instance, concerning resistance training, it can be noted that the American College of Sports Medicine (ACSM) suggests that novice practitioners engage in full-body workouts 2–3 times a week, intermediate participants partake in full-body workouts 3 times a week or adopt a split routine targeting upper and lower body 4 times a week, while advanced exercisers commit to 4–6 sessions weekly, each major muscle group trained once or twice a week (55).

Also it should be emphasized that in addition to the training seniority, there may be a relationship between training frequency and the strength of the habit, as indicated by the co-occurrence of performance frequency and stable connections as a key characteristic of the habit. Furthermore, previous data showed that since the habit measurement is constructed from the participants' answers to questions concerning frequency and stability, and has previously demonstrated good predictive validity and showing that frequency x stability measures of physical activity habits are highly related to the experience of physical activity as automatic (56). Therefore this reinforces the fact that there may be a relationship between the frequency of physical training and the formation of habits and the strength of the habit. Considering that the definition of habit formation in the APA Dictionary of Psychology is “ the process by which, through repetition or conditioning, animals or humans acquire a behavior that becomes regular and increasingly easy to perform” (57). Moreover, in the context of the assumption that veteran trainees, characterized as those engaged in physical activity for over 12 months, possess a more ingrained exercise habit, our findings substantiate the validity of this presumption. Therefore, when these findings are collectively synthesized, they imply a potential interplay between the strength of an individual's exercise habit, their duration as an exerciser, and the frequency of their training sessions. This study offers valuable insights into the intricate dynamics of exercise habits, seniority, and exercise frequency among participants, thus shedding light on the potential relationships within this multifaceted framework. When we examined the degree of adherence to physical activity in relation to the different types of training (Hypothesis 2), we discovered that those who reported that they trained in resistance training had the highest degree of adherence compared to aerobic training and flexibility training, but there were no significant differences found compared to training defined as “other training”. Furthermore, no statistically significant differences in training preference in regard to aerobic vs. resistance training were detected between men and women This contradicts previous research that found women prefer aerobic exercise over resistance training (31), that women have different cultural expectations about resistance training, and that women have higher levels of concern, all of which discourage them from participating in such training (58).

On the contrary, in the context of Hypothesis 3, wich predicted that the age and gender of the trainees would affect the goals of their physical training, it can be seen that the age of the trainees did affect the goal of the exercise, as trainees (both women and men) over the age of 45 preferred health goals more than younger trainees. We chose to investigate the issue of age because it is a significant risk factor for common diseases in affluent countries that includes cancer, cardiovascular disease and neurodegeneration (59). We also chose the age cut-off of 45 because, according to ACSM Risk Factor Screening, age is a risk factor that must be taken into account when approaching physical activity (60). Although age as a risk factor for men and women differs (men >45 years, women >55 years), we decided to perform use the age of 45 for both sexes. These findings are consistent with previous studies that found older people to be more concerned about their health than younger people and that physical activity was seen as an investment in health (36, 37). In contrast, younger adults under the average age of 45 were significantly motivated to exercise in order to keep their weight under control and to remain physically attractive (32). Moreover, the gender of the trainees exerts an influence on their exercise objectives. Notably, women tend to prioritize goals related to body toning and weight loss, while men gravitate toward objectives centered on muscle mass augmentation, enhanced physical fitness, and overall health improvement. This pattern of gender-related differences aligns with established empirical findings from prior research, underscoring the heightened significance of body weight concerns among women in comparison to men. Additionally, women tend to derive greater motivation from goals related to external appearance within the context of physical training, and their levels of concern about body weight, as well as the interplay of body composition, significantly impact their contentment and serve as motivational factors for engaging in exercise (25, 32, 34, 35). However, the trainees' seniority had no effect on the physical training goals, and while we predicted that veteran trainees would prefer goals related to health and improving physical fitness components over visibility and aesthetics goals compared to beginners, no significant statistical differences were shown.

When we predicted that the most veteran trainees over a year had received instruction and guidance in the past but were not currently receiving any (Hypothesis 5), interesting results were obtained when compared to the prediction. The veteran trainees reported that 40.65% of them had received such instruction in the past, but were not currently receiving any compared to beginners who reported that 38.89% of them were. However, it is possible that some of the beginners will still receive supervision later in their physical training journey, so this percentage may change. What is more intriguing are the findings regarding those who had received no instruction and guidance, with the percentage for veterans standing at 31.16% compared to 16.67% for beginners, and the fact that this figure may still change and decrease for beginners, as just mentioned. As a result, these findings may indicate that physical training adherence is related to an individual's capacity for self-efficacy, and it is possible that people who are more independent or who have gained stronger self-efficacy will stick more to long-term physical exercise over time. These findings are consistent with those of Ahern et al. (61), who point out that self-efficacy also has a significant effect on the motivation for physical activity. They found that there is a linear relationship between self-efficacy and attitudes towards physical activity and that as a person gains more experience in physical activity, his self-efficacy also increases. Furthermore, Gabay and Oravitan (12), suggest that in order to promote consistency and commitment, fitness instructors should strive to create a joyful and positive environment, offer challenges, and improve the self-efficacy of trainees. An additional assessment was performed to appraise the quality of life of the subjects in relation to their seniority, where in accordance to the prediction (Hypothesis 6), a statistically significant result was obtained between seniority and quality of life. These results make logical sense given that the current study's data show that veteran trainees exercise at a higher training frequency than novice trainees and that previous research found that people who exercised more frequently reported significantly better health and quality of life than those who exercised less frequently (62).

### Strengths

The participants in this study were primarily recruited through social media platforms. The phenomenal growth of such platforms has changed the ease of recruiting research participants, as they enable large groups of targeted participants to be quickly recruited at a low cost (63). In addition we chose to use an online survey because of the time required to complete it (data show that these surveys are two-thirds shorter than traditional ones), its ability to collect data automatically at a lower cost, to automatically and permanently save the database and access the data, and the fact that these surveys allow study participants to answer the survey questions at their choice of time and location (64). Further strengths of this investigation encompass the utilization of research instruments recognized for their robust reliability and internal consistency. For instance, the Self-Report Habit Index (SRHI) stands as a widely accepted and validated measure across diverse domains, particularly with regard to habit strength (65). Moreover, the Exercise Adherence Rating Scale (EARS) was purposefully crafted to assess adherence to physical activity, rendering it particularly germane to the study's focus (49). Notably, the World Health Organization Quality of Life (WHOQOL), developed by the esteemed World Health Organization (51), enjoys global acclaim and esteem as a comprehensive gauge of quality of life. It meticulously evaluates a multitude of domains, encompassing physical well-being, psychological health, social interactions, and environmental factors.

### Limitations

This study's strengths include its comprehensive analysis of many variables that might affect participants' adherence to physical exercise, its multiple hypothesis testing, and use of a large participant pool. However, it does have a number of obvious limitations. First, in relation to physical exercise, it should be noted that the definition of a habit as a particular behavior or action is oversimplified, as such exercise involves a complex variety of behaviors related to both the activity itself and the circumstances and actions that motivate an individual to engage in it such as planning, transportation, packing the necessary equipment, and financial ability (56). Moreover, in contrast to the preceding discussion, there are distinct drawbacks associated with the recruitment of research participants through social media. One salient issue pertains to the age demographics of active social media users, which predominantly lean toward younger individuals. Furthermore, ethnic and racial disparities in the utilization of social media platforms introduce a form of selection bias, as certain groups are more actively engaged than others. This selective engagement can further amplify the challenge of obtaining a representative study population (63). It is also essential to recognize that the characteristics of social media users may not accurately mirror the broader population. This discrepancy can potentially compromise the generalizability of research findings and introduce inherent biases within the study sample. Furthermore, in contrast to the aforementioned advantages, there are notable drawbacks associated with the utilization of online surveys as a research tool. While these surveys offer numerous benefits, they also have their limitations. One significant challenge pertains to the clarity of survey instructions, which, if ambiguous or poorly presented, can swiftly frustrate respondents, leading to premature survey abandonment. Additionally, online surveys rely heavily on self-reporting, which is susceptible to biases and inaccuracies (65).

Furthermore, it is imperative to address supplementary limitations warranting discussion. The observed outcomes pertaining to the influence of seniority on quality of life may, to some extent, be ascribed to considerations associated with the sample size. It is worth noting that these findings, while bearing statistical significance, manifest a relatively small in magnitude. Moreover, it is essential to duly acknowledge the presence of alternative variables that may exert a more pronounced impact on the quality of life experienced by individuals. Lastly, an imperative concern necessitates attention—the notable attrition rate among participants in the study, prominently manifested through instances of incomplete questionnaire responses.

## Conclusions

This study provides valuable insights into the challenging issue of adherence to physical activity, with implications for individual health and public well-being. The findings offer a fresh perspective on the factors influencing adherence, including training frequency, habit strength, exercise goals, age, gender, and support. Key practical recommendations include personalizing training programs, promoting resistance training, tailoring support based on age and gender, emphasizing habit development through frequent training, and boosting self-efficacy. Future interventions and research should focus on tailored exercise prescription, resistance training promotion, age and gender specific approaches, habit formation strategies, self-efficacy enhancement, long-term adherence monitoring, cultural considerations, and behavior change interventions to advance our understanding and faciltate exercise adherence.

**Practical Implications to Enhance Physical exercise Adherence:**

• **Personalized Training Programs:** It is advisable for healthcare professionals and fitness experts to prioritize personalized training programs tailored to each individual's distinct goals and preferences, as opposed to generic, one-size-fits-all programs.
• **Resistance Training:** Integrating resistance training into exercise routines is highly recommended. Resistance Training demonstrated notable adherence rates and may serve as an effective strategy to enhance individuals' commitment to physical activity.
• **Targeted Support:** Recognizing the influence of age and gender on exercise goals is essential. It is desirable for training regimens to be in alignment with the exerciser's age and gender. For instance, training programs for individuals over the age of 45 may need to place greater emphasis on health-related components, recognizing the unique needs of this demographic.
• **Training frequency:** Encouraging individuals to engage in training sessions exceeding three times a week may be a good strategy to cultivate and solidify physical exercise habit, ultimately improving long-term adherence.
• **Self-Efficacy Assessment:** It is desirable to identify the trainee's level of self-efficacy and preserve or increase it if necessary

## Data Availability

The raw data supporting the conclusions of this article will be made available by the authors, without undue reservation.
